# Synbiotics Containing Nanoprebiotics: A Novel Therapeutic Strategy to Restore Gut Dysbiosis

**DOI:** 10.3389/fmicb.2021.715241

**Published:** 2021-08-12

**Authors:** Liang Hong, Sang-Mok Lee, Whee-Soo Kim, Yun-Jaie Choi, Seo-Ho Oh, Yu-Ling Li, Seung-Hoon Choi, Dong Hyen Chung, Eunkyoung Jung, Sang-Kee Kang, Chong-Su Cho

**Affiliations:** ^1^Tianjin Key Laboratory of Agricultural Animal Breeding and Healthy Husbandry, College of Animal Science and Veterinary Medicine, Tianjin Agricultural University, Tianjin, China; ^2^Department of Agricultural Biotechnology, Seoul National University, Seoul, South Korea; ^3^Insilico Co., Ltd., Ansan-Si, South Korea; ^4^Research Institute of Agriculture and Life Sciences, Seoul National University, Seoul, South Korea; ^5^Institutes of Green-Bio Science & Technology, Graduate School of International Agricultural Technology, Seoul National University, Pyeongchang, South Korea

**Keywords:** nanoprebiotics, synbiotics, dysbiosis, gut microbiota, pathogenic infection, cross-feeding, gut barrier reinforcement, endotoxin

## Abstract

A new formulation, nanoprebiotics [e.g., phthalyl pullulan nanoparticles (PPNs)], was demonstrated to enhance the antimicrobial activity of probiotics [e.g., *Lactobacillus plantarum* (LP)] *in vitro* through intracellular stimulation better than that by backbone prebiotics, which are commonly used. In this study, we aimed to investigate whether this combination would exert distinct effects as synbiotics *in vivo*. Synbiotics combinations of LP, pullulan, and PPNs were used as experimental treatments in a dysbiosis-induced murine model, and their restorative effect was assessed using pathogen *Escherichia coli* K99 challenge. Our results showed that the *E. coli* infection was suppressed markedly in the experimental group fed with synbiotics containing PPNs. In addition, the decrease in serum endotoxin level after synbiotics treatment suggested the reinforcement of the gut barrier. Comparison of treatment groups, including a normal control group, showed that synbiotics containing PPNs increased microbial diversity, which is a representative parameter of healthy status. Furthermore, distinct from probiotics treatment alone, synbiotics showed additive effects of enrichment of several well-known beneficial bacteria such as *Lactobacillus, Bifidobacterium*, and other butyrate-producing bacteria including *Faecalibacterium*. Collectively, our results indicate that synbiotics containing PPNs are effective at restoring gut dysbiosis, suppressing pathogenic infection, and increasing microbial diversity, suggesting that synbiotics with nanoprebiotics have the potential to be a novel strategy for ameliorating gut dysbiosis and infectious diseases.

## Introduction

Considered by some researchers as a “forgotten organ,” the gut microbiota has attracted considerable attention in recent years, given its profound effect on host homeostasis (Espirito Santo et al., 2021). Indeed, several studies have substantiated that gut dysbiosis is highly associated with various diseases such as inflammatory bowel disease, obesity, and cancer (Guinane and Cotter, 2013; Aron-Wisnewsky et al., 2019; Fan et al., 2020). Therefore, maintaining or restoring gut microbiota in a balanced state is important for host health. Dysbiosis is often caused by the infection and proliferation of harmful microbes; therefore, the ability of gut microbiota to suppress its occurrence is very important for host health (Ducatelle et al., 2015). To solve this, several strategic therapies such as probiotics, prebiotics, and synbiotics are being widely studied (da Silva et al., 2021).

According to the World Health Organization, the Food Agriculture Organization, and the International Scientific Association for Probiotics and Prebiotics, probiotics are live microorganisms that provide health benefits to the host when adequate amounts are administered (Akour, 2020; Swanson et al., 2020). They are generally safe and can prevent and cure dysbiosis owing to their production of antibacterial peptides such as bacteriocins and their ability to enhance the intestinal barrier functions (Ohland and Macnaughton, 2010; Daba and Elkhateeb, 2020; Liu et al., 2020). In the case of prebiotics, they stimulate the growth of probiotics or other beneficial microorganisms in the gastrointestinal tract, decreasing pathogens and providing favorable effects to the host (Wang S. et al., 2020). Synbiotics, a combination of probiotics and prebiotics, are also designed to produce a synergistic effect on pathogen suppression (Zheng et al., 2021). In particular, various mechanisms of synbiotics have been proposed such as improving the viability or functionality of probiotics in the gastrointestinal tract by selective utilization of their synbiotics partner (e.g., prebiotics) (Swanson et al., 2020). Moreover, there is a concept that synbiotics may be complementary synbiotics, where each component is working independently, although they all aim to produce benefits to the host and make it easy for gut microbiota to control pathogens by strengthening their antimicrobial ability.

Our previous studies demonstrated that nanoprebiotics (NPs), whose backbones were inulin, dextran, starch, and pullulan, increased the antimicrobial ability of probiotics *in vitro* (Kim et al., 2018, 2019; Hong et al., 2019, 2020). They enhanced the expression of bacteriocin biosynthetic genes and activated the defense system of the probiotics through internalizing within the probiotics. The probiotics showed extremely high antimicrobial activity against both gram-positive and gram-negative pathogens when treated with NPs. In the case of dextran, an *in vivo* feeding experiment in a normal mouse model was conducted to investigate the effects on gut microbiota when fed with the NPs and their probiotics partner (Kim et al., 2019). Although PDN was tested only in eubiosis conditions, the study implied that synbiotics with NPs had the potential to be preventive drugs against gut dysbiosis. However, their effects under dysbiosis conditions have not been investigated yet. Moreover, the previous study did not investigate the effects of synbiotics comprising a mixed form of prebiotics backbone and NPs together as components of synbiotics, although synbiotics using NPs showed distinct benefits compared to the case of backbone for the synbiotic partners.

In this study, we aimed to determine whether synbiotics containing NPs are effective at recovering from dysbiosis in terms of restoring the gut barrier and suppressing pathogenic infection. Accordingly, phthalyl pullulan nanoparticles (PPNs) were prepared using pullulan, and a newly designed synbiotics combination (probiotics: *Lactobacillus plantarum* (LP), prebiotics: pullulan, and PPNs) was treated to an antibiotics-induced gut dysbiosis murine model. Subsequently, the dysbiosis-ameliorating effects were evaluated by measuring the amount of invading pathogen in the host gut and monitoring alterations in the gut microbiome and other host physiological changes after pathogen challenges.

## Results

### Synthesis and Characterization of PPNs

**FIGURE 1:**
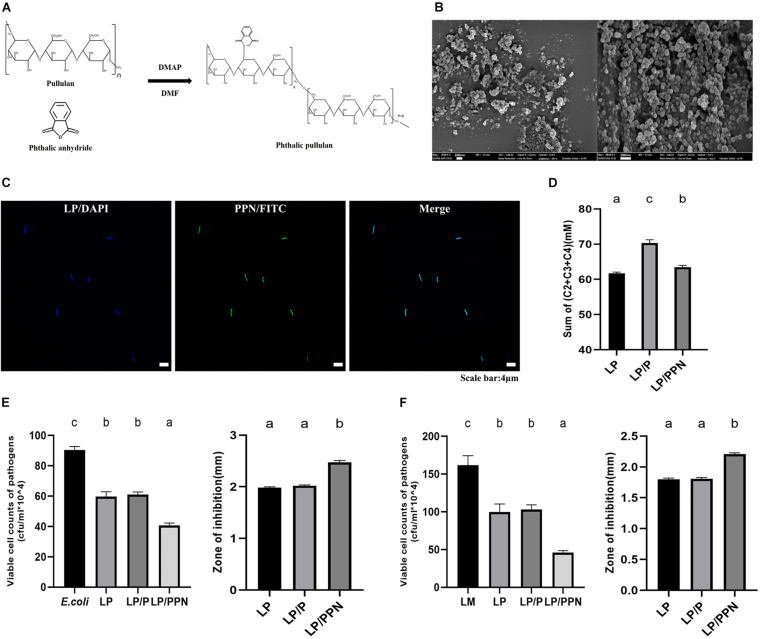
Synthesis and characterization of PPNs. Chemical reaction scheme for the synthesis of PPNs **(A)**. Morphologies of PPNs were observed by scanning electron microscopy (SEM; magnification: 100.00k, scale bar = 200 nm) **(B)**. Analysis of the internalization of PPNs into LP observed by confocal laser scanning microscopy (CLSM) **(C)**. The sum of SFCA amount when culturing probiotics LP alone (LP) or treated with pullulan (LP/P) or PPNs (LP/PPN) was measured using gas chromatography **(D)**. Antimicrobial activity of probiotics LP when treated with pullulan (LP/P) or PPNs (LP/PPN) against *Escherichia coli* (EC) **(E)** and LM **(F)**. One-way ANOVA with Tukey’s *post hoc* test was used to determine significant differences among groups. Different superscript letters indicate statistical significance (*p* < 0.05).

The chemical reaction scheme of phthalyl pullulan is shown in Figure 1A. Following the same method as previously reported, the content of phthalate groups in phthalyl pullulan was confirmed by^1^ H-nuclear magnetic resonance (NMR) spectroscopy measurement and was estimated by determining the ratio of phthalic acid protons to sugar protons (Hong et al., 2019). Observation under a scanning electron microscope indicated that the morphologies of the PPNs were spherical as shown in Figure 1B. The internalization of PPNs was confirmed by confocal laser scanning microscopy. As shown in Figure 1C, the PPNs were internalized into LP.

To examine any internal changes in LP by PPNs, LP was treated with PPNs or pullulan, and its commonly secreted short chain fatty acids (SFCAs), such as acetate (C2) (Supplementary Figure 1A), propionate (C3) (Supplementary Figure 1B), and butyrate (C4) (Supplementary Figure 1C), were analyzed *in vitro*. It was found that the total SCFA amount (mM) in the culture medium of LP (61.70 ± 0.36) was changed by a lesser extent when treated PPNs (63.46 ± 0.51) than when treated with pullulan (70.32 ± 0.93) (Figure 1D).

The antimicrobial activities of the PPN-internalized LP against pathogen *Escherichia coli* (Figure 1E) and *Listeria monocytogenes* (LM) (Figure 1F) were evaluated using the coculture assay (CFU/ml × 10^4^) and agar diffusion test (mm). In *E. coli*, the results for the coculture assay (*E. coli*: 90.33 ± 2.31, LP: 59.67 ± 3.21, LP/P: 61.00 ± 1.73, and LP/PPN: 40.67 ± 1.53) and agar diffusion test (LP: 1.98 ± 0.02, LP/P: 2.02 ± 0.02, and LP/PPN: 2.48 ± 0.03) showed distinguished antibacterial ability in the PPN-treated groups. Likewise, similar patterns were observed in both the coculture assay (LM: 161.67 ± 12.58, LP: 99.67 ± 10.50, LP/P: 103.00 ± 6.08, and LP/PPN: 46.00 ± 2.65) and agar diffusion test (LP: 1.80 ± 0.02, LP/P: 1.81 ± 0.02, and LP/PPN: 2.21 ± 0.02) using LM as the pathogen. Both cases showed that the antimicrobial activity of the PPN-internalized LP was much higher than that of untreated LP or pullulan alone.

### Physiological Changes in Host

**FIGURE 2:**
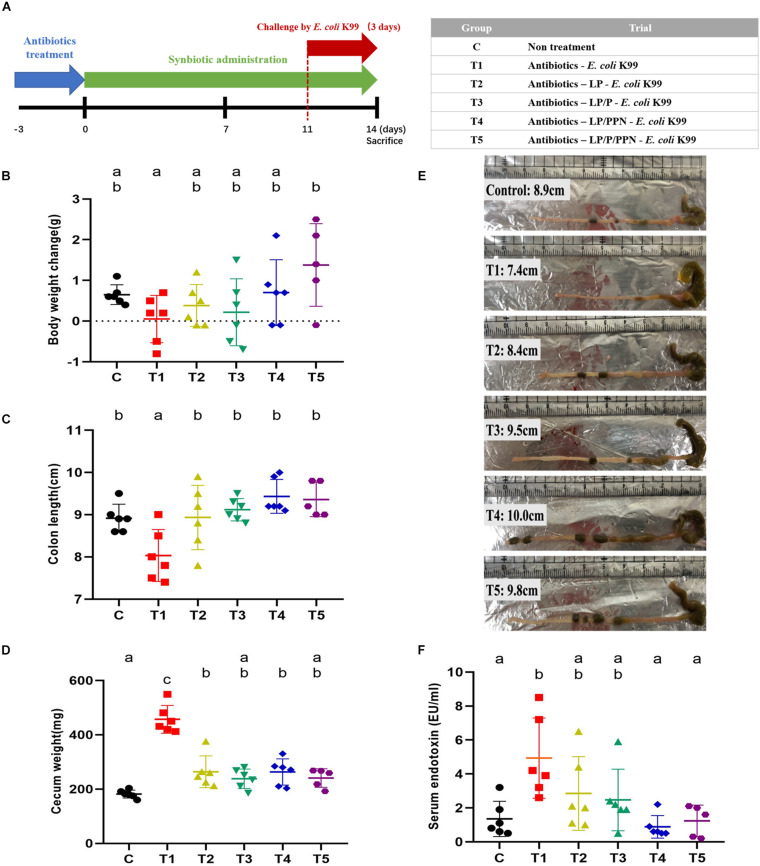
*In vivo* experiment schedule and physiological changes of dysbiosis-induced mice upon pro-/synbiotics after pathogen *Escherichia coli* infection. Experiment schedule and group information **(A)**. Individual body weight changes from start to end of the experimental period in each group **(B)**. Colon length **(C)** and cecum weight **(D)** were measured at the end of the experiment, and their representative picture after dissection **(E)**. Serum endotoxin (LPS) level per group was measured using sera samples **(F)**. One-way ANOVA with Tukey’s *post hoc* test was used to determine significant differences among groups. Different superscript letters indicate statistical significance (*p* < 0.05).

As shown in Figure 2A, the mouse feeding experiment was performed to evaluate the effect of synbiotics against dysbiosis. The effectiveness of antibiotic treatment in mimicking dysbiosis was determined by the extent of decrease in colonies on the Luria-Bertani (LB) agar compared to that in a saline-treated group (Supplementary Figure 2).

The greatest body weight change (g) at the end of the feeding experiment was observed for the T5 group (1.38 ± 1.01), which was supplemented with the LP/P/PPN combination, whereas the body weight of the T1 group (0.05 ± 0.58) was decreased at the endpoint (Figure 2B; C: 0.65 ± 0.24; T2: 0.38 ± 0.52; T3: 0.22 ± 0.82; and T4: 0.70 ± 0.81). Average feed intake values per mouse were numerically higher in the PPN-supplemented groups (T4 and T5) than in the others groups (Supplementary Figure 3).

There were also significant changes in colon length (cm) and cecum weight (g) with pro-/synbiotics treatment. The T4 group (9.43 ± 0.40) had the longest colon length, and all groups supplemented with pro-/synbiotics had lengths similar to that of the C group (8.92 ± 0.33) and had longer colon length than that of the T1 group (8.03 ± 0.62) (Figure 2C; T2: 8.93 ± 0.76, T3: 9.12 ± 0.26, and T5: 9.36 ± 0.41; Figure 2E), showing that the pattern was similar to that observed in the body weight. Likewise, a noticeable increase in the cecum weights was only observed in the T1 (451.17 ± 51.30) group, to whom only *E. coli* was administered, whereas the other groups fed with probiotics or synbiotics, especially the T3 (238.17 ± 35.74) and T5 (240.80 ± 34.13) groups, showed low cecum weights similar to that of the C group (Figure 2D).

To assess gut barrier restoration by pro-/synbiotics, the levels of serum endotoxin (EU/ml) were measured. The T1 group (4.93 ± 2.36) fed only with *E. coli* showed the highest value, whereas the levels were significantly lower in the T4 (0.88 ± 0.66) and T5 (1.24 ± 0.92) groups fed with LP/PPN and LP/P/PPN, respectively (Figure 2F; C: 1.35 ± 1.04; T2: 2.85 ± 2.17; and T3: 2.47 ± 1.81). Likewise, with the injection of fluorescein isocyanate (FITC)–dextran (μg/ml) into the guts of mice to determine intestinal permeability, a similar tendency was observed; the T1 group (1.69 ± 0.57) showed the highest level of FITC–dextran in serum, whereas significantly lower levels were observed in the synbiotics-fed groups, including T4 (0.59 ± 0.40) and T5 (0.50 ± 0.20) (Supplementary Figure 4; C: 0.75 ± 0.26; T2: 1.04 ± 0.33; and T3: 0.61 ± 0.29).

### Effects of Synbiotics on the Gut Microbiota

To determine the effects of PPNs on gut microbiota, both culture-independent and -dependent analyses were performed using intestinal contents and their genomic DNA (gDNA).

**FIGURE 3:**
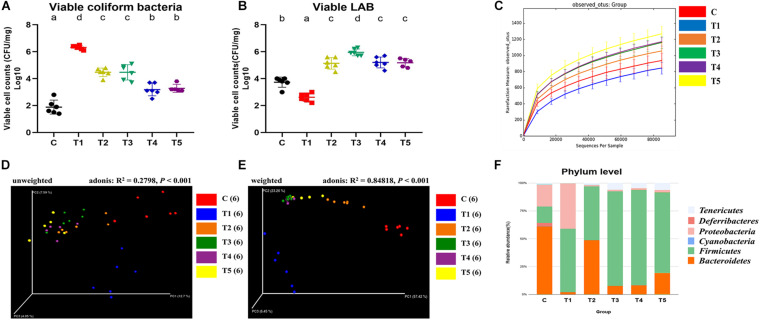
Effects of pro-/synbiotics on the gut microbiota of murine dysbiosis model with pathogen *Escherichia coli* infection. The viable cell counts of coliform bacteria **(A)** and LAB **(B)** were enumerated by plating 10-fold serially diluted intestinal contents (original concentration: 10 mg intestinal contents/1 ml PBS) onto MacConkey agar and MRS agar, respectively. QIIME version 1.9.1 software was used to investigate alpha diversity indices such as microbial richness (observed OTUs) per group **(C)**, PCoA plot based on unweighted **(D)**, and weighted **(E)** UniFrac distances, and the overall compositions of the gut microbiota at the phylum level **(F)**. One-way ANOVA with Tukey’s *post hoc* test was used to determine significant differences among groups. Different superscript letters indicate statistical significance (*p* < 0.05).

First, viable cells (log_10_ CFU/mg) of coliform bacteria, including *E. coli* and lactic acid bacteria (LAB) were enumerated by plating intestinal contents onto MacConkey agar and De Man, Rogosa, and Sharpe (MRS) agar, respectively. The mean log value of viable coliform bacteria of the T1 group (6.32 ± 0.15) was approximately 6 log_10_, whereas that of the other groups treated with pro-/synbiotics (T2: 4.47 ± 0.31, T3: 4.47 ± 0.57, T4: 3.18 ± 0.46, and T5: 3.28 ± 0.295) were significantly lower (Figure 3A). Interestingly, the PPN-treated groups (T4 and T5) showed a significant decrease in coliform bacteria compared to that in the T2 and T3 groups, although not as much as in the C group (1.88 ± 0.52).

In contrast, the LAB amounts in groups tended to be contrary to the results for coliform. The mean log value of LAB amount for the T1 group (2.62 ± 0.30) was significantly the lowest among groups, whereas the values of pro-/synbiotics groups (T2: 5.13 ± 0.43, T3: 5.95 ± 0.25, T4: 5.20 ± 0.40, and T5: 5.18 ± 0.31) were all significantly higher (Figure 3B). In addition, the values of those groups were significantly increased compared to that of the C group (3.73 ± 0.38).

To cross-check the results of culture-dependent analysis and investigate overall changes in the gut microbial community by pro-/synbiotics, gDNA-based analyses such as quantitative polymerase chain reaction (qPCR) and 16S rRNA sequencing were performed using species-specific primers (Supplementary Table 1). With qPCR (fold change), similar results were observed in the levels of enteropathogenic *E. coli* (intimin) (Supplementary Figure 5A; C: 1.10 ± 0.10, T1: 662.67 ± 217.49, T2: 4.17 ± 0.15, T3: 4.98 ± 0.94, T4: 2.78 ± 0.64, and T5: 3.07 ± 0.45) and LAB, especially *Lactobacillus* spp. (Supplementary Figure 5B; C: 1.09 ± 0.10, T1: 0.52 ± 0.03, T2: 4.63 ± 0.50, T3: 20.98 ± 5.05, T4: 9.48 ± 0.72, and T5: 11.50 ± 1.44) and *Bifidobacterium* spp. (Supplementary Figure 5C; C: 1.44 ± 0.41, T1: 1.35 ± 0.43, T2: 2.93 ± 0.73, T3: 3.40 ± 0.27, T4: 6.57 ± 0.50, and T5: 4.07 ± 0.15).

Next, microbial community dynamics were explored based on 16S rRNA sequencing. Observed operational taxonomic units (OTUs), a microbial richness index, were high in the order of T1, C, T2, T3, T4, and T5 (Figure 3C). Other alpha diversity indices such as Shannon (diversity index; Supplementary Figure 6A; C: 5.72 ± 0.27, T1: 3.02 ± 0.45, T2: 5.53 ± 0.52, T3: 6.73 ± 0.19, T4: 6.63 ± 0.24, and T5: 6.88 ± 0.52) and Simpson (evenness index; Supplementary Figure 6B; C: 0.94 ± 0.01, T1: 0.70 ± 0.09, T2: 0.91 ± 0.03, T3: 0.97 ± 0.01, T4: 0.97 ± 0.01, and T5: 0.97 ± 0.02) had similar patterns wherein the lowest value was commonly observed in T1 and the highest in T5. To examine the effect of PPNs as synbiotic partners on microbial richness, the groups were reorganized by PPN treatment (C,T1,T2,T3 vs. T4,T5). Interestingly, observed OTUs were high when treating with PPNs (Supplementary Figure 6C).

Principal coordinate analysis (PCoA) was performed based on unweighted (Figure 3D; *R*^2^ = 0.28, *p* < 0.001) and weighted (Figure 3E; *R*^2^ = 0.85, *p* < 0.0001) UniFrac distances and the Adonis test. The results, especially in weighted UniFrac distances, revealed that the gut microbiota was altered by synbiotics treatment. In the PCoA plot, samples were clustered into three distinct groups (C vs. T1 vs. T2, T3, T4, and T5). The samples of T2, T3, T4, and T5 were placed between the C and T1 samples, each of which was also distinguished from the other.

Next, the relative abundance of microbial taxa in each group was compared, and it was found that several phyla and genera appeared to be at quite different levels. At the phylum level, all groups shared the following 13 phyla: Actinobacteria, Bacteroidetes, Cyanobacteria, Deferribacteres, Euryarchaeota, Firmicutes, Fusobacteria, Lentisphaerae, Proteobacteria, Spirochetes, TM7, Tenericutes, and Verrucomicrobia (Supplementary Table 2). The three dominant phyla, containing more than 95% of the total 16S rRNA gene sequences, were Bacteroidetes, Firmicutes, and Proteobacteria in the C and T1 groups, whereas in the T2, T3, T4, and T5 groups, these were Bacteroidetes, Firmicutes, and Tenericutes (Figure 3F). In particular, Proteobacteria was more abundant in the T1 group than in the C group, whereas it was significantly reduced in the T2, T3, T4, and T5 groups (Supplementary Figure 7).

At the genus level, the gut microbiota of the six groups shared 102 genera (Supplementary Table 2). Three dominant genera containing more than 55% of the total 16S rRNA gene sequences were (1) C group: an unclassified genus of family *S24-7*, *Helicobacter*, and *Odoribacter*; (2) T1 group: unclassified genera of families Enterobacteriaceae, Erysipelotrichaceae, and, Lachnospiraceae; (3) T2 group: *Oscillospira*, unclassified genera of families Lachnospiraceae and Rikenellaceae; (4) T3 and T4 groups: *Oscillospira*, an unclassified genus each of families Lachnospiraceae and Ruminococcaceae; and (5) T5 group: unclassified genera of families Lachnospiraceae, Rikenellaceae, and Ruminococcaceae (Supplementary Figure 8A). In particular, *Lactobacillus* was more abundant in T4 and T5 than in other groups (Supplementary Figure 8B). Likewise, *Bifidobacterium* was significantly more abundant in T4 fed with LP/PPN than in the other groups, which is similar to the results of qPCR (Supplementary Figure 8C). In addition, *Faecalibacterium* and the unclassified genus of the family Veillonellaceae showed significantly higher abundance in the T5 group than in the other groups (Supplementary Figures 8D,E).

Taken together, supplementing synbiotics, especially LP/PPN or LP/P/PPN, modulated gut microbiota by increasing microbial richness and diversity. Concurrently, the relative abundances of several bacteria were differed among the groups.

### Predicted Effects of Synbiotics on the Gut Metagenome

To predict the functions of the gut metagenome of each group, the abundances of Kyoto Encyclopedia of Genes and Genome (KEGG) pathways were predicted using the PICRUSt software, and prediction accuracy was assessed using the Nearest Sequenced Taxon Index (NSTI) score. The average NSTI scores of C, T1, T2, T3, T4, and T5 were 0.18 (±0.02), 0.07 (±0.03), 0.12 (±0.01), 0.17 (±0.01), 0.15 (±0.01), and 0.15 (±0.01), respectively, which were similar to those reported in other mammal microbiota studies (Langille et al., 2013; Kieler et al., 2019). Subsequently, linear discriminant analysis (LDA) effect size (LEfSe) analysis was performed to determine KEGG pathways whose abundances were different among groups.

**FIGURE 4:**
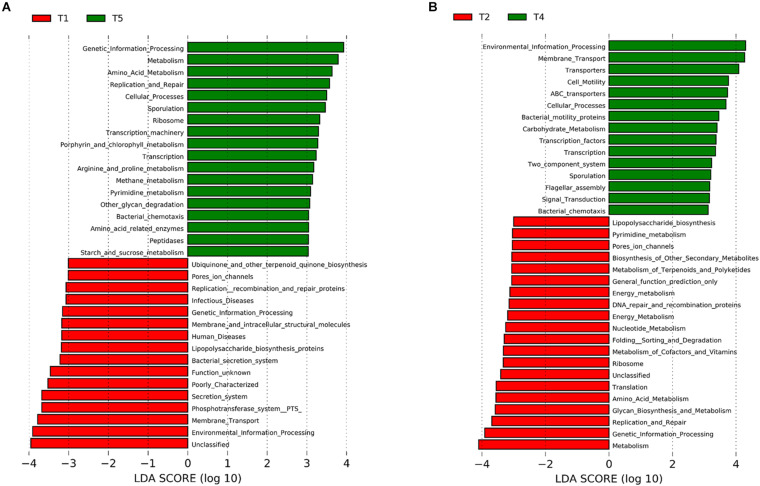
Metagenomic prediction of gut microbiota of dysbiosis-induced mice upon pro-/synbiotics after pathogen *Escherichia coli* infection. Microbial functions were predicted using PICRUSt at the third level of the KEGG pathway. LEfSe analysis was represented as histogram determining synbiotics LP/P/PPN effect (LDA score > 3.0) **(A)** and NP PPN effect (LDA score > 3.0) **(B)**.

The effect of synbiotics on the KEGG pathways was predicted by comparing the T1 and T5 groups. It was found that several significantly different KEGG pathways were identified between the two groups (Figure 4A). For example, “metabolism,” “amino acid metabolism,” “replication and repair,” and “cellular processes” were predicted at significantly higher levels in the T5 group, whereas “infectious diseases,” “lipopolysaccharide biosynthesis,” “bacterial secretion system,” and “membrane transport” were predicted at significantly higher levels in the T1 group. A similar result was observed when comparing T1 with T4 (Supplementary Figure 9A).

The effect of PPNs on the intestinal microbiome was also predicted by comparing the T2 and T4 groups (Figure 4B). Between the two, the T4 group showed higher levels for “membrane transport,” “transporters,” “ABC transporters,” “carbohydrate metabolism,” “transcription factors,” and “transcription,” whereas the T2 group showed higher levels for “lipopolysaccharide biosynthesis,” “pore’s ion channels,” “folding sorting and degradation,” and “glycan biosynthesis and metabolism.”

## Discussion

In this study, we report perturbation in gut microbiota and host physiology associated with 2-week supplementation with synbiotics, including PPNs. Distinct from the case of PDN (NP whose backbone was dextran; Kim et al., 2019) in the previous study, which was conducted under eubiosis condition, synbiotics, including PPNs, were administered to a *in vivo* antibiotics-induced dysbiosis murine model. In addition, pullulan was also used as a synbiotic partner to investigate whether it could provide additive benefits to host health. Subsequently, the ability of these pro-/synbiotics to restore the gut from dysbiosis and improve susceptibility against pathogens was evaluated by how effectively they suppressed infection of pathogen EC.

First, PPNs were prepared and examined for their ability to increase the antimicrobial ability of LP *in vitro* as described in a previous study (Hong et al., 2019). Furthermore, it was observed that pullulan can be fermented by LP to produce SCFAs, which are crucial for intestinal health. Especially, butyrate is well known for regulating the intestinal barrier function, and the role of propionate in alleviating dextran sulfate sodium-induced intestinal dysfunction has been demonstrated (Tong et al., 2016; Bach Knudsen et al., 2018). Compared to LP alone, when treated with PPNs, although there was no significant increase in the level of acetate (mM; Supplementary Figure 1A; LP: 61.60 ± 0.36, LP/P: 67.37 ± 0.81, and LP/PPN: 61.70 ± 0.44), the levels of propionate (μM; Supplementary Figure 1B; LP: 98.67 ± 2.08, LP/P: 1683.67 ± 95.82, and LP/PPN: 766.00 ± 18.33) and butyrate (μM; Supplementary Figure 1C; LP: non-detected, LP/P: 1268.33 ± 56.59, and LP/PPN: 993.67 ± 102.71) were slightly increased. However, when compared to the results of LP/P, in LP/PPN, all SCFA values (acetate, propionate, and butyrate) were significantly low. This implied that PPNs could function as particles themselves when internalized into probiotics, not as carbon sources for microbial fermentation.

Moreover, there were significant changes in microbial richness, diversity, and gut composition, and improvements in host physiological indices following the synbiotics-supplementing trial, which supported the dysbiosis-restoring effect of synbiotics. The increases in body weight and feed intake of synbiotics, especially with LP/PPN and LP/P/PPN, showed the ability of synbiotics to recover the pathogen-suppressing activity of gut microbiota, as weight and appetite losses are both common signs of inflammation triggered by pathogenic *E. coli* (Greenhill, 2016). These results suggest that synbiotics containing PPNs may prevent weight loss and intestinal inflammation, which are provoked by pathogenic infection. Furthermore, colon length and cecum weight are typically used to determine whether the gut is in an abnormal state, as they are the main reservoirs of intestinal microbes. It was reported that the colon became short when it was damaged by pathogen invasion or inflammation (Kajiya et al., 2009). In the case of the cecum, its swelling was observed in antibiotics-administered mice and similar to that seen in the case of germ-free mice (Okada et al., 1994; Nameda et al., 2007). In this study, *E. coli* infection induced decreases in colon length and increases in cecum weight, which were both restored to the normal state by synbiotics treatment. These results suggest that synbiotics with NPs may play an important role in ameliorating gut disruption through strengthening the gut barrier. Indeed, the gut permeability was improved as the influx of endotoxin into the blood circulation was limited by synbiotics supplementation, especially that of LP/PPN and LP/P/PPN. As endotoxin is widely known to provoke endotoxemia, causing inflammation and various diseases, this result implies that synbiotics, including NPs, may have the potential to be novel therapeutics to treat endotoxemia or its associated diseases (Thorn, 2001; Moludi et al., 2020).

In terms of the microbiome, there were several interesting findings suggesting that synbiotics can also be useful in rebuilding disrupted gut microbiota. Notably, the viable cell count of challenged pathogens was monitored to determine the degree of gut recovery robustness by pro-/synbiotics. As expected, synbiotics with NPs showed a distinguished ability to inhibit more pathogens compared to probiotics alone or synbiotics without NPs. In addition, synbiotics increased the number of beneficial bacteria such as LAB in the gut. The results of gene expression levels detecting enteropathogenic *E. coli* (e.g., intimin), *Lactobacillus* spp., and *Bifidobacterium* spp., by qPCR of bacterial gDNA using intestinal contents showed similar results as that of their counterparts of viable cell counts, supporting that synbiotics may provide the benefits of both suppressing pathogens and promoting commensal beneficial bacteria. Furthermore, synbiotics influenced changes not only in certain species but also in overall microbial communities of gut microbiota. Numerous studies have shown that the diversity of gut microbiota is one of the principal features of health status and host health (Backhed et al., 2012; Claesson et al., 2012; Jacouton et al., 2017). In this study, the alpha diversity indices such as the Shannon index (Supplementary Figure 6A), Simpson index (Supplementary Figure 6B), and observed OTUs (Supplementary Figure 6C) were consistently the highest in the microbiota of synbiotics LP/P/PPN-treated groups, whereas it was the lowest in the group that was only infected with the pathogen. Consistent with results of previous studies, the results of alpha diversity indices obtained in this study support that synbiotics containing NPs may be functioning to maintain gut microbiota in the normal state against dysbiosis. In the PCoA plot based on weighted UniFrac distances, distinct clusters were observed with pathogen infection and pro-/synbiotics treatment. Considering these results, it can be seen that synbiotics with NPs cause an apparent shift in the gut microbiota in a positive manner, which offset the adverse effects of dysbiosis and pathogen invasion, and even improvement in key indices to more than that observed in the normal state.

In addition to externally invading pathogens, there are numerous commensal bacteria with opportunistic pathogenicity already distributed in the intestine (Bhat et al., 2019). For example, Proteobacteria, which was significantly decreased when treated with synbiotics, is the phylum that contains many disease-provoking bacteria such as *Escherichia*, *Vibrio*, *Helicobacter*, and *Salmonella* (Litvak et al., 2017). As the pathogen *E. coli* used in this study is also one of the species that belongs to Proteobacteria, the decrease in the relative abundance of this phylum by synbiotics treatment suggests that synbiotics may have restrained excessive growth of pathogens in the gut.

At the genus level, various genera were also affected by synbiotics treatment (Supplementary Figure 8; Supplementary Table 2). Interestingly, distinct from the treatment with probiotics alone, pullulan and PPNs showed additive effects by enriching several commensal beneficial bacteria. For example, the relative abundances of both *Lactobacillus* (Supplementary Figure 8B) and *Bifidobacterium* (Supplementary Figure 8C), which are widely used owing to their probiotic properties, increased in the microbiota, which corresponded to the qPCR results (Suez et al., 2020). Moreover, the relative abundances of several butyrate-producing bacteria such as *Faecalibacterium* (Supplementary Figure 8D), the unclassified genus of the family Veillonellaceae (Supplementary Figure 8E), *Coprococcus*, and *Ruminococcus* were increased when treated with synbiotics, especially LP/P/PPN (Supplementary Table 2). These bacteria have attracted attention because of their distinguished ability to produce butyrate, which plays important roles in gut homeostasis, including energy source for colonocytes and immunomodulatory factors (Zeng et al., 2019). In particular, *Faecalibacterium* has been reported as a representative biomarker of healthy microbiota (Cheema, 2019). One of the genera in the family Veillonellaceae, *Veillonella*, was reported for its abundance in a physically active state (Scheiman et al., 2019). Likewise, *Coprococcus* and *Ruminococcus* were reported as beneficial bacteria owing to their anticancer effect against colon cancer and decreased levels in various microbiota-related diseases such as inflammatory bowel disease, respectively (Nagao-Kitamoto and Kamada, 2017; Ai et al., 2019).

Based on PICRUSt analysis, several significant differences were observed, although the findings should be interpreted with caution. In mice fed with synbiotics LP/PPN or LP/P/PPN, the genes related to nutrient metabolism and normal cellular processes were prevalent, whereas the pathways for bacterial invasion and the associated diseases were scarce. Furthermore, the gene related to lipopolysaccharide (LPS) biosynthesis was also predicted to be upregulated by *E. coli* infection. Furthermore, there were intriguing functional differences when NPs (PPNs) worked as a synbiotic partner. For example, compared to probiotics alone, when treating with NPs, the pathways representing membrane transport or transcription were upregulated, whereas the pathway for LPS biosynthesis was downregulated. In addition, distinct from when using the backbone polymer (pullulan) as prebiotics, NPs upregulated pathways representing glycan synthesis and various carbohydrate metabolism. These results suggest that synbiotics with NPs may recover gut microbiota from damage mainly by promoting metabolism regarding various nutrients and inhibiting LPS biosynthesis. In particular, considering a decrease in endotoxin (LPS) levels with synbiotics treatment was observed in this study, the result may be consistent with the hypothesis on the LPS biosynthesis-suppressing effect of synbiotics, although further *in vivo* studies are required because this has not been shown as a cause-and-effect relationship to date.

As LPS is a representative component of endotoxins originating from gram-negative bacteria such as *Proteobacteria*, we chose to examine whether there were bacteria whose relative abundance in the gut was correlated with serum endotoxin levels (Supplementary Figure 10; Rizzatti et al., 2017). Expectedly, *Proteobacteria* (*r* = 0.45, *p* = 0.007; Supplementary Figure 10A) was the only phylum positively correlated with serum endotoxin level, although no phylum showed a negative correlation. In addition, several genera such as *Cronobacter* (*r* = 0.62, *p* < 0.001; Supplementary Figure 10B), unclassified genus of the Enterobacteriaceae family (*r* = 0.60, *p* < 0.001; Supplementary Figure 10C), *Enterobacter* (*r* = 0.59, *p* < 0.001; Supplementary Figure 10D), and *Streptococcus* (*r* = 0.49, *p* = 0.003; Supplementary Figure 10E) were positively correlated. *Cronobacter* has been known to provoke severe illnesses such as necrotizing enterocolitis, meningitis, and septicemia in people with a vulnerable gut, including neonates, infants, and the elderly (Holý and Forsythe, 2014). *Streptococcus* is widely studied as a pathogen provoking various diseases (Wu et al., 2014). Several genera that belong to the Enterobacteriaceae family, including *Enterobacter*, are regarded as fatal pathogens because of their resistance to antibiotics and relation with diseases (Davin-Regli et al., 2019). However, beneficial bacteria such as *Lactobacillus* (*r* = −0.34, *p* = 0.046) (Supplementary Figure 10F), which was increased by synbiotics, was negatively correlated with serum endotoxin level. Therefore, it is likely that synbiotics LP/P/PPN modulated gut microbiota by mainly inhibiting LPS-producing bacteria, thus contributing to the alleviation of endotoxin influx into the host circulation system.

Overall, both the microbiological and physiological findings suggest that a novel form of synbiotics containing NPs has distinguished effects on restoring dysbiosis, compared to those of probiotics or synbiotics alone with backbone polysaccharides. The novel synbiotics demonstrated the potential for inducing gut barrier reinforcement by modulating gut microbiota, ultimately providing physiological benefits to the host.

Specifically, in the case of gut microbiota, it may be possible to effectively inhibit pathogens in *Proteobacteria* such as coliform bacteria by administering probiotics, whose expression of bacteriocin might be increased by NPs when considering the *in vitro* results. Although the levels of intestinal SCFA have not been monitored, probiotics themselves would have made further effects such as SCFA production by fermenting the pullulan backbone while inhabiting the gut. In particular, an increase in butyrate, which is known as a nutrient source for colonocytes (Fu et al., 2019), may promote colonocyte proliferation and inhibit abnormal gut changes such as colon shrinkage and cecum swelling caused by dysbiosis. In addition, various cross-feeding interactions among probiotics and commensal gut microbes could help recover intestinal homeostasis. For example, cross-feeding between *Lactobacillus* and *Bifidobacterium* (Moens et al., 2017), also between *Bifidobacterium* and butyrate-producing bacteria (Turroni et al., 2018; Kim et al., 2020), has been reported. The increasing levels of *Bifidobacterium* and various butyrate-producing bacteria such as *Faecalibacterium*, *Veillonellaceae*, *Coprococcus*, or *Ruminococcus* may also be attributed to the administration of *Lactobacillus* and its cross-feeding. Given that the *Faecalibacterium* is one of the most widely studied bacteria, as well as an important indicator and contributor to intestinal health (Ferreira-Halder et al., 2017), its increase has supported the dysbiosis-restoring effect of synbiotics. Besides, as the lactate-utilizing bacteria group has been proposed as important for the stability of the gut microbiota system (Wang S. P. et al., 2020), an increase of lactate in the gut lumen by administering LAB may also have enhanced the stability of the system. Indeed, other representative indices to gauge intestinal health such as microbial richness, evenness, and diversity also showed that the microbial system recovered to a normal state.

Taken together, the novel synbiotics can suppress pathogen more effectively than probiotics alone on account of the improvement of bacteriocin-producing ability by NPs and SCFA-producing ability by backbone polysaccharide, while encouraging cross-feeding among probiotics and various commensal bacteria. As a result, the novel synbiotics may restore the gut microbiota composition back to the similar state as the eubiosis condition. Considering various changes in physiological parameters, it can be inferred that the effects of synbiotics on gut microbial dynamics can reinforce the gut barrier and give beneficial impacts on the host. Reduced gut permeability has been demonstrated by decreased levels of circulating LPS and FITC–dextran, although the expression of the colonocyte tight junction such as ZO-1 and occludin was not directly monitored. Combined with the results of metagenome prediction suggesting inhibition of LPS biosynthesis in the synbiotics-treated group, synbiotics may strengthen the gut barrier and inhibit the inflow of microbe-associated molecular patterns (MAMPs) such as LPS from the gut lumen to the host systemic circulation system. Therefore, synbiotics may alleviate MAMP-triggered inflammation, which acts as a cause of many diseases. Furthermore, the correlation results among circulating LPS levels and the relative abundance of each gut microbe revealed that decreased circulating LPS levels by synbiotics may inhibit the impact of disease-provoking pathogens such as *Proteobacteria*, *Cronobacter*, *Enterobacter*, and *Streptococcus*. Finally, the changes in gut microbiota and the resulting gut barrier reinforcement would have positively affected the host phenotypic parameters. Indeed, various pathogen-mediated adverse events such as suppressing body weight gain, shrinking colon, and swelling cecum have all been ameliorated by administering the novel synbiotics. More studies are required to clarify the effects on the gut microbiota and hosts when only treated with NPs without a probiotics partner. In addition, to be used as a microbiome drug, there should be a process of verifying that these novel synbiotics can act as effectively as in this study in various enteropathic disease models. Nevertheless, the results suggest that NPs may have the potential to be a novel agent as a synbiotics partner and can therefore be widely applicable to improve susceptibility to invasive pathogens and to cure diseases associated with dysbiosis such as inflammatory bowel disease.

## Materials and Methods

### Materials

Pullulan used in this study was purchased from Shandong Freda Biotechnology Co., Ltd. (Shandong, China), and other chemicals were purchased from Sigma-Aldrich (St. Louis, MO, United States). For bacterial cultures, LB broth, LB agar, MRS broth, brain heart infusion (BHI) broth, MacConkey sorbitol agar, and Oxford agar were purchased from BD Difco (Sparks, MD, United States).

### SCFA Production of Synbiotics

To determine the availability of SCFAs through pullulan and PPNs by LP, *in vitro* SCFA profiles of LP when fermenting pullulan and PPNs were investigated. Gas chromatography was performed according to a previously described method with a slight modification (Erwin et al., 1961). Briefly, a 5.0-ml aliquot of cultured supernatant was mixed with 1.0 ml of 25% metaphosphoric acid and 0.2 ml of 2% pivalic acid (internal standard), and the mixture was analyzed using an Agilent 7890B gas chromatography system (Agilent Technologies, Santa Clara, CA, United States) with an flame ionization detector (FID). The inlet and detector temperature were maintained at 220°C. Aliquots (1 μl) were injected with a split ratio of 10:1 into a 30 m × 0.25 mm × 0.25 μm Nukol fused-silica capillary column (cat. no.: 24107, Supelco, Sigma-Aldrich) with helium carrier gas set to a flow rate of 1 ml/min. The oven temperature was held constant at the 80°C for 1 min, and thereafter increased at 20°C/min to a temperature of 180°C and held for 1 min, and increased at 10°C/min to a final temperature of 200°C. The total run time per sample was approximately 14 min.

### Synthesis of PPNs

Phthalyl pullulan was synthesized according to the fine-tuned method described in the previous study (Hong et al., 2019). In brief, 1.0 g of pullulan was dissolved in 10 ml of dimethylformamide, and 24 mg of dimethylamino pyridine (0.1 mol/mol sugar residues of pullulan) was added to the solution as a catalyst. Subsequently, 2.7 g of phthalic anhydride was added to the above solution at a 9:1 (phthalic anhydride:pullulan) molar ratio. The reaction was performed at 54°C for 48 h under nitrogen. The produced phthalyl pullulan was dialyzed first in dimethylformamide to remove unreacted phthalic anhydride and then in distilled water at 4°C for 24 h to form self-assembled PPNs. The unreacted pullulan was removed after ultracentrifugation. Finally, the PPNs were freeze dried and stored at −20°C until further use. The surface topography of the PPNs was analyzed using field emission scanning electron microscopy with 55VP-SEM (Carl Zeiss, Oberkochen, Germany).

### *In vitro* Evaluation of Antimicrobial Activity

To determine the effects of PPNs on the antimicrobial activity of LP, the coculture assay and agar diffusion test were conducted using *E. coli* and LM as pathogens as described previously with slight modifications (Ditu et al., 2011; Driscoll et al., 2012). LP, *E. coli*, and LM were cultured in MRS, LB, and BHI broth, respectively, under 37°C with shaking (255 rpm) for 24 h before being used in subsequent experiments or being stored at −70°C in 15% (v/v) glycerol.

For the coculture assay, 2.0 × 10^6^ CFU/ml of *E. coli* or LM was cocultured with 2.0 × 10^5^ CFU/ml of LP treated with or without 0.5% (w/v) PPNs or pullulan in MRS broth for 8 h at 37°C under aerobic conditions in a shaking incubator (250 rpm). The cocultured samples were spread on MacConkey or Oxford agar and incubated for 24 h at 37°C, and the numbers of *E. coli* or LM colonies were counted, respectively. For the agar diffusion assay, 100 μl of *E. coli* or LM stock (2.0 × 10^8^ CFU/ml) was spread onto LB or BHI agar. A paper disc was placed on the pathogen-spread plate. Then, 120 μl of 8-h-cultured media of LP treated with or without (0.5% w/v) PPNs or pullulan was dropped onto the paper disc. After drying at room temperature, the plate was cultured overnight at 37°C. The zones of inhibition were used as a direct measurement of antimicrobial activity.

### Animal Experimental Procedures and Measurements

The synbiotic feeding study was performed using 4-week-old BALB/c female mice following international ethical guidelines. The Institutional Animal Care and Use Committee (IACUC) at Seoul National University approved the animal experiments (SNU-180904-2-1). Mice were housed at a controlled temperature (22 ± 2°C) on a 12-h light/dark cycle. Animals were fed a standard mouse chow diet and provided distilled water *ad libitum*. After 7 days of acclimation, mice were randomly allocated into six groups (six BALB/c mice per group, one cage per group) (Figure 2A). During the experiment period, all mice were tracked individually by marking identification numbers on their tails. The control group (C) continued to be fed as before without any antibiotics, synbiotics, and pathogen challenge during the overall experimental period. The other groups (T1–T5) were administered an antibiotics cocktail (ampicillin:gentamicin:neomycin:vancomycin = 2:2:2:1, total 20 mg/mice) for 3 days at the beginning of the experiment to induce dysbiosis in their gut according to previously described methods with modification (Samuelson et al., 2017; Bayer et al., 2019).

Fecal samples were spread onto LB agar and colonies numerated to simply check the effect of antibiotics on gut microbiota (Supplementary Figure 2). Subsequently, the five groups, except the C group, were fed with pro-/synbiotics, which were resuspended in 200 μl of saline solution and administered via oral gavage for 2 weeks. Before resuspension, the pro-/synbiotics were prepared by mixing lyophilized powder of each probiotic or prebiotics in precalculated amounts. Treatments per group were as follows: (1) T1 (no pro-/synbiotic), (2) T2 (LP 10^8^ CFU/mice), (3) T3 [LP 108 CFU mixed with 0.5 wt.-% pullulan/mice (LP/P)], (4) T4 [LP 108 CFU mixed with 0.5 wt.-% PPNs/mice (LP/PPN)], and (5) T5 [LP 10^8^ CFU mixed with 0.5 wt.-% pullulan and 0.5 wt.-% PPNs/mice (LP/P/PPN)].

From the 11th day of feeding pro-/synbiotics, *E. coli* (10^9^ CFU/mice) was administered with 0.2 ml of 1% NaHCO_3_ (treated 30 min before *E. coli* administration) to the mice from the T1 to T5 groups via oral gavage for 3 days. When administering probiotics LP or *E. coli*, the numbers were controlled by enumerating their CFUs using agar plating and calculating the amount for 10^8^ CFU and 10^9^ CFU before administration, respectively.

The body weights and food intakes of mice were monitored daily over the entire experimental period. At the end of the experiment, mice were sacrificed using CO_2_. Then, ceca, colons, intestinal contents, and sera samples were collected for further analysis. Intestinal contents were collected in the form of a mixture of cecum and colon contents of each mouse at the time of sacrifice. Blood samples (2 ml/mice) were collected via retro-orbital bleeding, and serum samples were isolated by centrifugation at 12,000 rpm for 3 min using the serum separator tube (BD microtainer, United States). The colon lengths and the weight of ceca samples were measured immediately after dissection.

Viable CFU counts of lactobacillus and coliform bacteria were enumerated using phosphate-buffered saline (PBS)-resuspended intestinal contents (10 mg intestinal contents/ml PBS). Then, 100 μl of resuspended samples were serially diluted 10-fold and spread onto MRS and MacConkey sorbitol agar, respectively, followed by incubation for 20 h at 37°C.

### *In vivo* Gut Permeability Assay

To evaluate the pro-/synbiotic effect on gut barrier integrity, the FITC–dextran assay and serum endotoxin level detection kit were used (Chelakkot et al., 2017). On the day of sacrifice, mice were subjected to fasting for 4 h, followed by intragastric injection with FITC–dextran tracer (0.6 mg/g body weight; cat. no. 46944, Sigma-Aldrich) dissolved in 0.1 ml of PBS. After 3 h, sera samples were collected to measure the intensity value of FITC fluorescence at an excitation wavelength of 490 nm and an emission wavelength of 530 nm using a Hitachi F-4500 fluorescence spectrophotometer. The samples were diluted in PBS to plot a standard curve. The measurement was obtained a minimum of three times per sample. Serum endotoxin (LPS) levels were detected using the Pierce^TM^ Chromogenic Endotoxin Quant Kit (Thermo Fisher Scientific, Waltham, MA, United States) according to the protocol of the manufacturer. Diluted serum samples (50 μl) were tested using a colorimetric method.

### DNA Extraction and Sequencing

The bacterial gDNA of intestinal samples were extracted to identify and estimate specific bacteria or their community using qPCR and high-throughput sequencing of the bacterial 16S ribosomal RNA (16S rRNA) genes.

DNA was extracted according to the protocol of the manufacturer from 50 mg of each intestinal contents sample using the AccuPrep^®^ Stool DNA extraction kit (Bioneer, Daejeon, South Korea), followed by storage at −20°C until further analysis. For species-specific qPCR, the primers used were designed based on the DNA sequences of bacteria of interest for detection, and qPCR was performed as previously described (Brown et al., 2016). The primers used to detect LAB (*Lactobacillus* and *Bifidobacterium*) and *E. coli* are listed in Supplementary Table 1.

To explore the microbial community of intestinal samples, the V4 region of the bacterial 16S rRNA gene was amplified using Takara Ex-Taq polymerase (Takara Bio, Shiga, Japan) and universal primers (F: 5′ GTGCCAGCMGCCGCGGTAA-3′, R: 5′-GGACTACHVGGGTWTCTAAT-3′). The amplification program was as follows: 1 cycle of 94°C for 3 min, 40 cycles of 94°C for 45 s, 55°C for 1 min, and 72°C for 1.5 min, and 1 cycle of 72°C for 10 min. The amplicons were separated by agarose gel electrophoresis (100 V, 45 min) and purified using a QIAquick Gel Extraction Kit (Qiagen, Valencia, CA, United States).

The NEB Next Ultra DNA Library Prep Kit for Illumina (New England Biolabs, Ipswich MA, United States) was used to construct DNA libraries according to the instructions of the manufacturer with some modification. The size selection steps for the adaptor-ligated DNAs and the cleanup steps were replaced by PCR products using a QIAquick PCR Purification Kit (Qiagen, CA, United States). The adaptor and index primers were added to the amplicons using the NEBNext Multiplex Oligos for Illumina Kit (New England Biolabs). The construction of DNA libraries was confirmed by agarose gel electrophoresis, and the libraries were purified using a QIAquick Gel Extraction Kit. The components of the libraries were then sequenced using an Illumina MiSeq 2 × 250 base pair (bp) paired-end sequencing platform (Macrogen, Daejeon, South Korea). The 16S rRNA gene sequences determined in this study were deposited in the NCBI SRA database with accession number SRR11341465.

### Microbial Community Analysis

The microbial community was analyzed using Quantitative Insights Into Microbial Ecology (QIIME) version 1.9.1 software and several in-house Perl scripts. Briefly, raw sequence reads were checked for their quality by FastQC V0.11.8 and trimmed by FASTX-Toolkit v.0.0.13 before the poor-quality region. Then, trimmed paired-end reads were merged using FLASH v1.2.11 and demultiplexed. The sequence reads were then clustered into OTU tables by subsampled open-reference OTU picking at a 97% level of sequence similarity with the GreenGenes 13_8 database as the reference. The OTU picking method was usearch61, and the value of parameter percent_subsample was 0.1 (Edgar, 2010). The representative sequences were aligned using the PyNAST program, which was taxonomically assigned using the uclust consensus taxonomy assigner (Desantis et al., 2006).

The microbial diversity of the samples (alpha diversity) was determined using the abundance-based coverage estimator, Chao1, observed OTUs, phylogenetic diversity, Shannon, and Simpson indices. These indices were calculated from 85,000 sequence reads through rarefaction with 10 iterations. Principal coordinates analysis was performed at the phylum and genus levels based on weighted and unweighted UniFrac distances, and the effects of probiotics, PPNs, pullulan, or their synbiotics were evaluated using Adonis statistical tests with the “vegan” package in R. The abundance of the microbial taxa was expressed as a percentage of the total 16S rRNA gene sequences.

### Prediction of the Functions of the Microbial Communities

The Phylogenetic Investigation of Communities by Reconstruction of Unobserved States (PICRUSt) version 1.0.0^1^ was used to predict the functional profile of the microbial communities based on the 16S rRNA gene sequences obtained (Douglas et al., 2018). The metagenomes were predicted using the precalculated KEGG orthologs and classified in a hierarchy using the KEGG pathway metadata. LDA was performed using LefSe at *p* < 0.05 and LDA > 3.0 using Galaxy^2^ (Segata et al., 2011).

### Statistical Analysis

One-way ANOVA and *post hoc* Tukey’s honestly significant difference (HSD) test for multiple mean comparisons were used to find significant differences among groups. One-way ANOVA and simple linear regression were performed using R statistics and “corrplot” packages version 3.0.3 (R Foundation for Statistical Computing, Vienna, Austria), respectively. The significance was assumed at ^∗^*p* < 0.05, ^∗∗^*p* < 0.01, and ^∗∗∗^*p* < 0.001.

To explore the relationship between serum endotoxin level and intestinal microbiota, simple linear regression analysis was performed, and significance was assessed by Pearson’s correlation coefficient (r) and *p*-values.

## Data Availability Statement

The original contributions presented in the study are publicly available. This data can be found here: https://www.ncbi.nlm.nih.gov/sra/?term=SRR11341465.

## Ethics Statement

The animal study was reviewed and approved by the Institutional Animal Care and Use Committee (IACUC) at Seoul National University approved the animal experiments (SNU-180904-2-1).

## Author Contributions

LH designed and conducted the experiments and wrote the manuscript. S-ML analyzed the data and wrote the manuscript. W-SK, S-HO, and Y-LL performed the animal feeding experiments. S-HC, DC, and EJ discussed the results and corrected the manuscript. Y-JC, S-KK, and C-SC supervised the project. All authors contributed to the article and approved the submitted version.

## Conflict of Interest

S-ML, S-HC, DC, and EJ are employed by Insilico Co., Ltd. The remaining authors declare that the research was conducted in the absence of any commercial or financial relationships that could be constructed as a potential conflict of interest.

## Publisher’s Note

All claims expressed in this article are solely those of the authors and do not necessarily represent those of their affiliated organizations, or those of the publisher, the editors and the reviewers. Any product that may be evaluated in this article, or claim that may be made by its manufacturer, is not guaranteed or endorsed by the publisher.

## Footnotes

1
http://picrust.github.io/picrust/

2
https://huttenhower.sph.harvard.edu/galaxy/

## Supplementary Material

Supplementary Figure 1 *In vitro* SCFA fermentation profile of probiotics LP upon treating prebiotics pullulan or nanoprebiotics phthalyl pullulan nanoparticles (PPNs). The levels of acetate **(A)**, propionate **(B)**, and butyrate **(C)** in the culture medium of LP were measured in triplicate using gas chromatography.

Supplementary Figure 2 Relative ratio of colony counts on Luria-Bertani (LB) agar after mice were treated with antibiotics (ampicillin:gentamicin:neomycin:vancomycin = 2:2:2:1, total 20 mg/mice) was compared to that of a saline-treated group. Each sample was plated on LB agar in triplicate after 10-fold serial dilution.

Supplementary Figure 3 Average daily feed intake per mouse over the experimental period.

Supplementary Figure 4 Levels of fluorescein isocyanate (FITC)–dextran in sera samples of each group after intragastric injecting FITC–dextran tracer. The FITC–dextran tracer (0.6 mg/g body weight) was dissolved in 0.1 ml of phosphate-buffered saline. The blood level of FITC-dextran was monitored in triplicate by measuring the intensity value of FITC fluorescence in sera samples at an excitation wavelength of 490 nm and an emission wavelength of 530 nm to investigate the degree of gut permeability in mice.

Supplementary Figure 5 Fold changes in the gene expression of markers detecting the enteropathogenic *Escherichia coli* (intimin) **(A)**, *Lactobacillus* spp. **(B)**, and *Bifidobacterium* spp. **(C)** in samples of intestinal contents by group. All samples were measured in triplicate. Detailed sequence information for each marker is listed in Supplementary Table 1.

Supplementary Figure 6 Differences in the gut microbial diversity among groups upon pro-/synbiotics treatment. Alpha diversity indices (**A:** Shannon index; **B:** Simpson index) of gut microbiota by group. Effects of phthalyl pullulan nanoparticle (PPN) treatment on observed operational taxonomic units (OTUs) **(C)**. All alpha diversity indices were investigated using QIIME version 1.9.1 software.

Supplementary Figure 7 Relative abundance of *Proteobacteria* by group. One-way ANOVA with Tukey’s *post hoc* test was used to determine significant differences among groups, and different superscript letters indicate a significant difference (*p* < 0.05).

Supplementary Figure 8 Overall compositions of the gut microbiota at the genus level **(A)** and relative abundance of genera significantly differed by group. **(B)** One-way ANOVA with Tukey’s *post hoc* test was used for each genus to determine significant differences among groups, and different superscript letters indicate a significant difference (*p* < 0.05).

Supplementary Figure 9 Metagenomic prediction of gut microbiota of dysbiosis-induced mice after the trial to determine the effects of synbiotics LP/phthalyl pullulan nanoparticles (LP/PPN) (**A**; T1 vs. T4, linear discriminant analysis (LDA) score > 3.0) or prebiotics nanoparticulation (**B**; T3 vs. T4, LDA score > 2.0). Microbial functions were predicted using PICRUSt at the third level of the KEGG pathway, and LEfSe analysis was represented as histograms.

Supplementary Figure 10 Correlation analysis between the level of serum endotoxin and relative abundance of bacteria. *Proteobacteria* **(A)** was the only phylum that showed a correlation with serum endotoxin. Several genera, including *Cronobacter* **(B)**, unclassified genus of the Enterobacteriaceae family **(C)**, *Enterobacter* **(D)**, and *Streptococcus* **(E)**, were significantly positively correlated, whereas *Lactobacillus* **(F)** was significantly negatively correlated with the level of serum endotoxin. The relationship was assessed by Pearson’s correlation coefficient (r) and *p*-values from simple linear regression.

Supplementary Table 1 Primer sequences to identify specific bacteria [*Escherichia coli* (*intimin*), *Lactobacillus* spp., *Bifidobacterium* spp., and 16S rRNA (*B16S*)] were used to conduct quantitative polymerase chain reaction (qPCR) with gDNA of intestinal contents.

Supplementary Table 2 Relative abundance of phyla and genera of the mouse ceca microbiota and all-sharing taxon upon pro-/synbiotics treatment. Data are shown as mean ± SD rounded off to the third digit after the decimal point. One-way ANOVA with Tukey’s *post hoc* test was used to determine significant differences among groups. Different superscript letters indicate statistical significance (*p* < 0.05).
