# Supplementary figures and images for: Synbiotics Containing Nanoprebiotics: A Novel Therapeutic Strategy to Restore Gut Dysbiosis
Source: Front Microbiol. 2021 Aug 12;12:715241. doi: 10.3389/fmicb.2021.715241 (PMC8406803; doi:10.3389/fmicb.2021.715241)

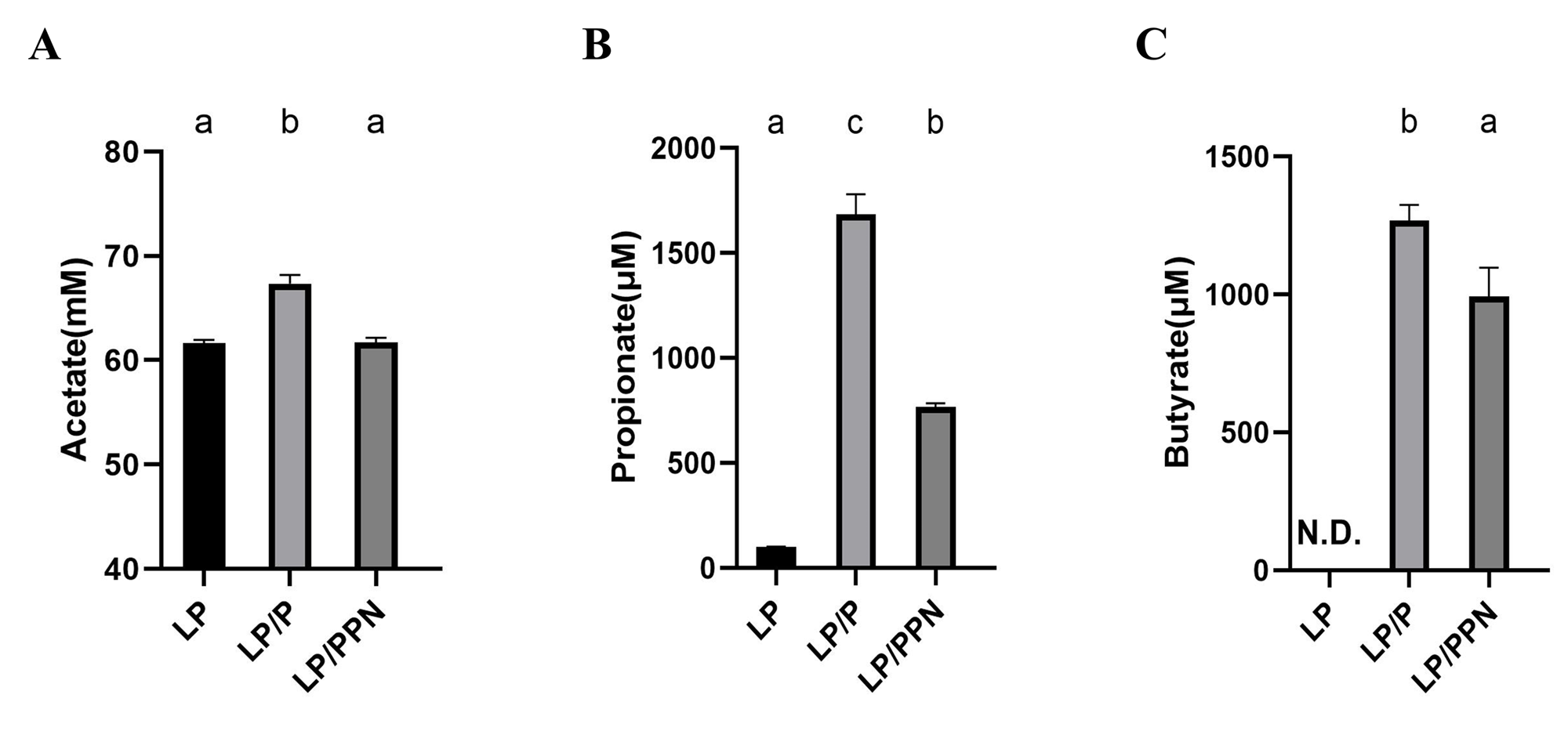

Supplement: Supplementary Figure 1 — In vitro SCFA fermentation profile of probiotics LP upon treating prebiotics pullulan or nanoprebiotics phthalyl pullulan nanoparticles (PPNs). The levels of acetate (A), propionate (B), and butyrate (C) in the culture medium of LP were measured in triplicate using gas chromatography. [file Image_1.TIF]

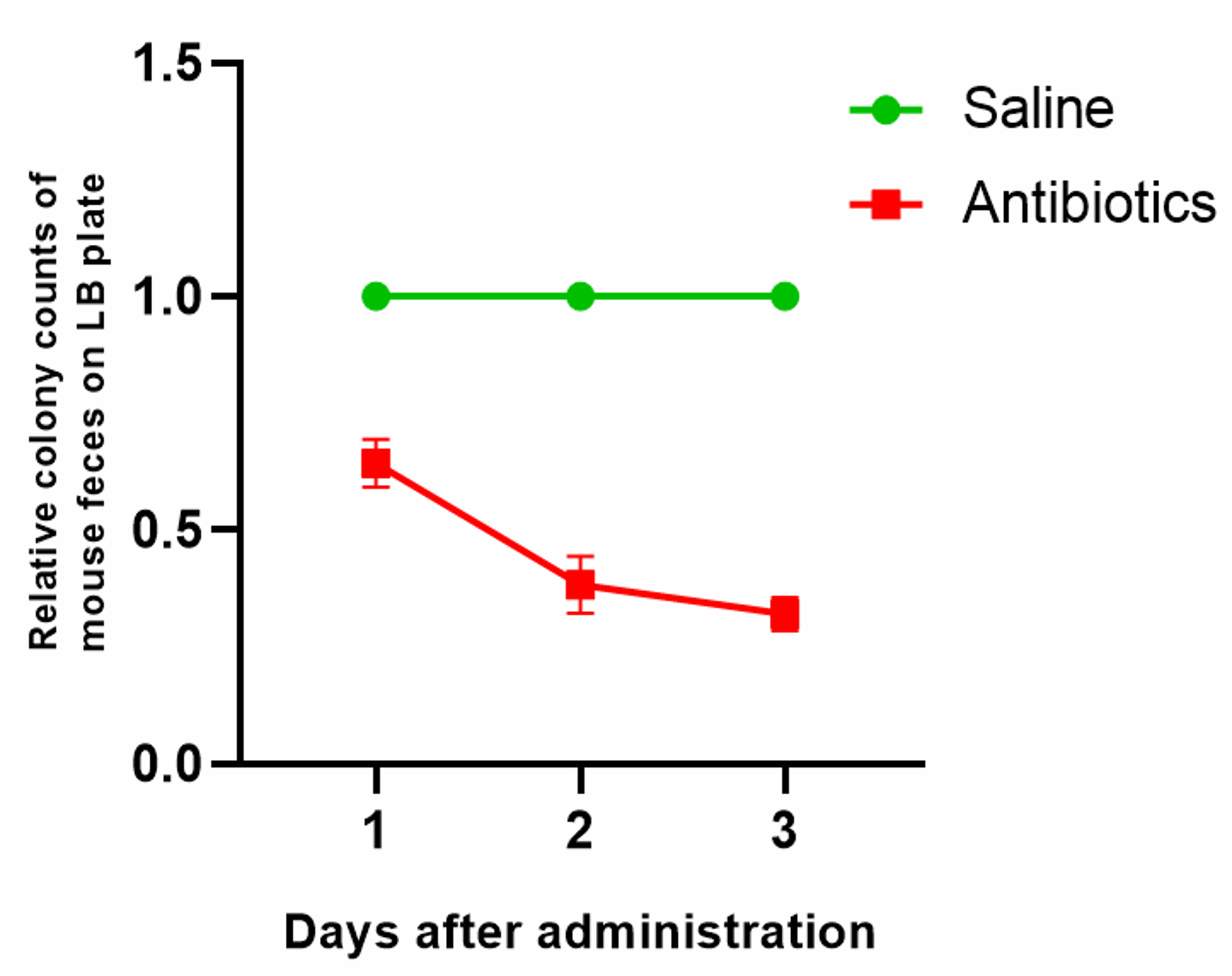

Supplement: Supplementary Figure 2 — Relative ratio of colony counts on Luria-Bertani (LB) agar after mice were treated with antibiotics (ampicillin:gentamicin:neomycin:vancomycin = 2:2:2:1, total 20 mg/mice) was compared to that of a saline-treated group. Each sample was plated on LB agar in triplicate after 10-fold serial dilution. [file Image_2.TIF]

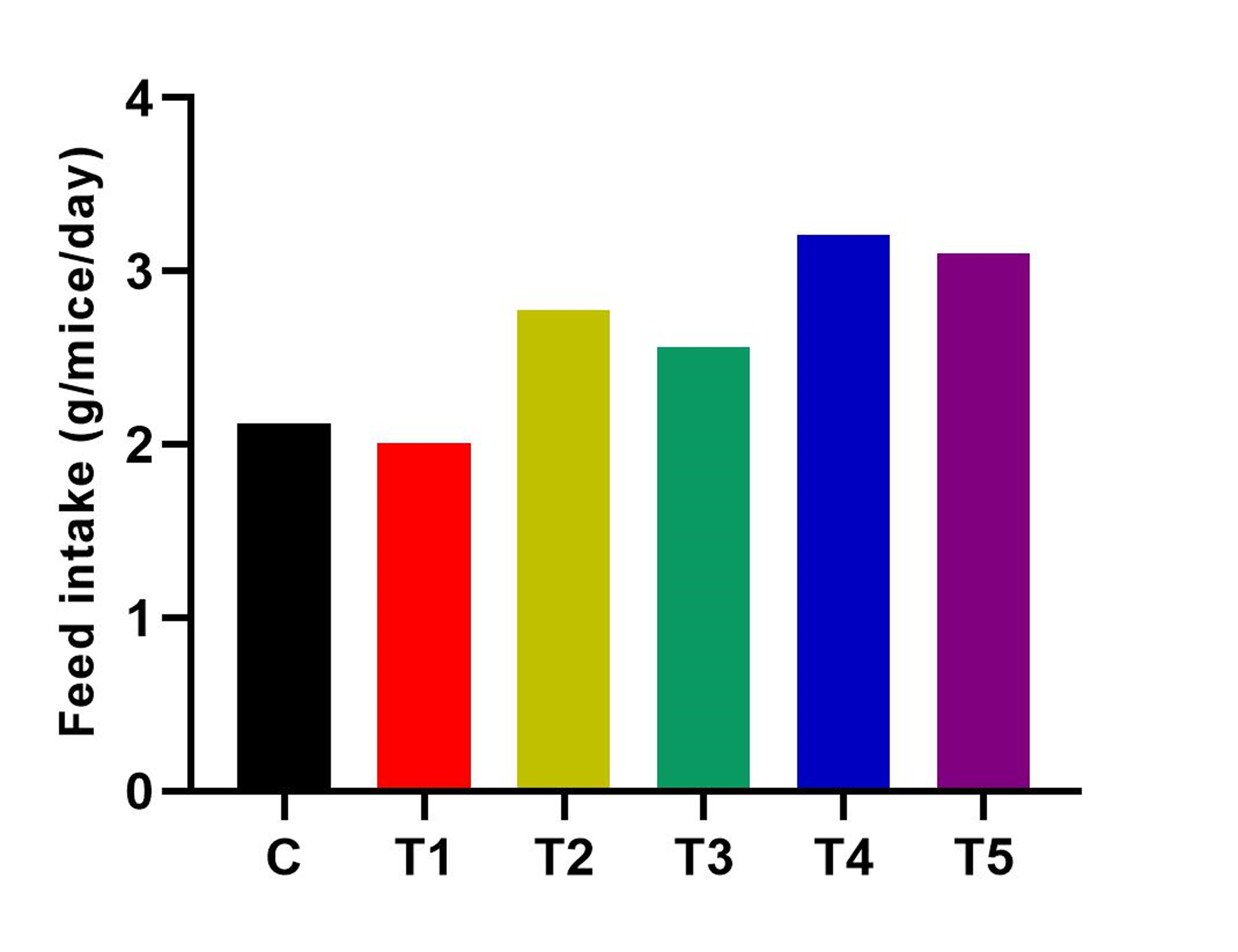

Supplement: Supplementary Figure 3 — Average daily feed intake per mouse over the experimental period. [file Image_3.TIF]

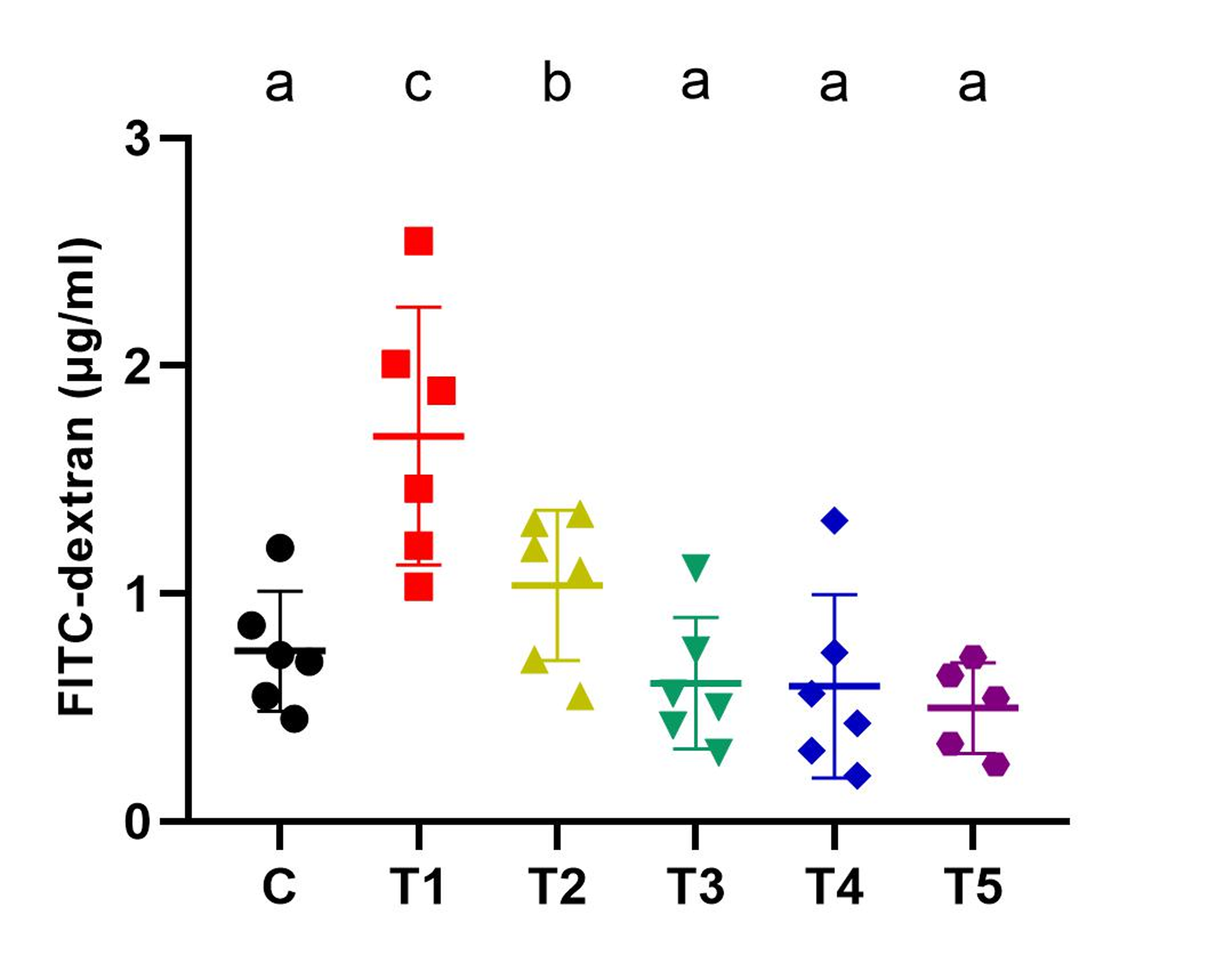

Supplement: Supplementary Figure 4 — Levels of fluorescein isocyanate (FITC)–dextran in sera samples of each group after intragastric injecting FITC–dextran tracer. The FITC–dextran tracer (0.6 mg/g body weight) was dissolved in 0.1 ml of phosphate-buffered saline. The blood level of FITC-dextran was monitored in triplicate by measuring the intensity value of FITC fluorescence in sera samples at an excitation wavelength of 490 nm and an emission wavelength of 530 nm to investigate the degree of gut permeability in mice. [file Image_4.TIF]

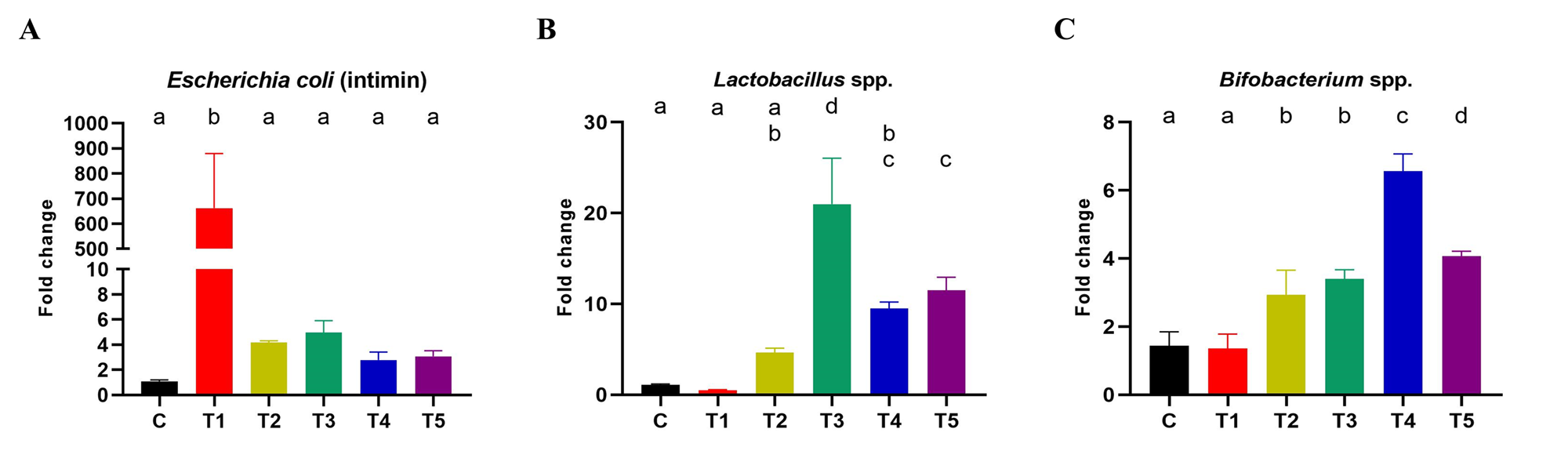

Supplement: Supplementary Figure 5 — Fold changes in the gene expression of markers detecting the enteropathogenic Escherichia coli (intimin) (A), Lactobacillus spp. (B), and Bifidobacterium spp. (C) in samples of intestinal contents by group. All samples were measured in triplicate. Detailed sequence information for each marker is listed in Supplementary Table 1. [file Image_5.TIF]

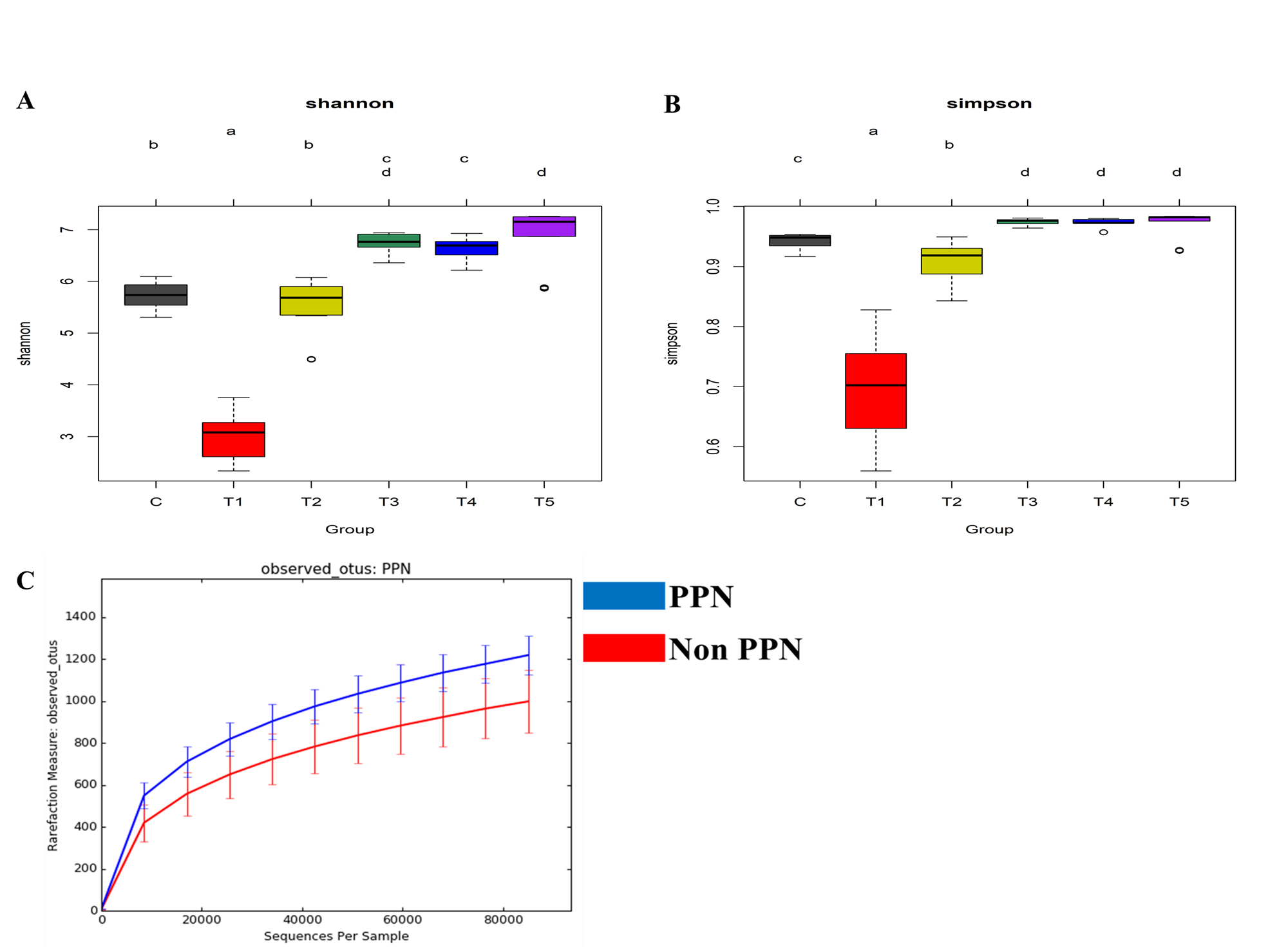

Supplement: Supplementary Figure 6 — Differences in the gut microbial diversity among groups upon pro-/synbiotics treatment. Alpha diversity indices (A: Shannon index; B: Simpson index) of gut microbiota by group. Effects of phthalyl pullulan nanoparticle (PPN) treatment on observed operational taxonomic units (OTUs) (C). All alpha diversity indices were investigated using QIIME version 1.9.1 software. [file Image_6.TIF]

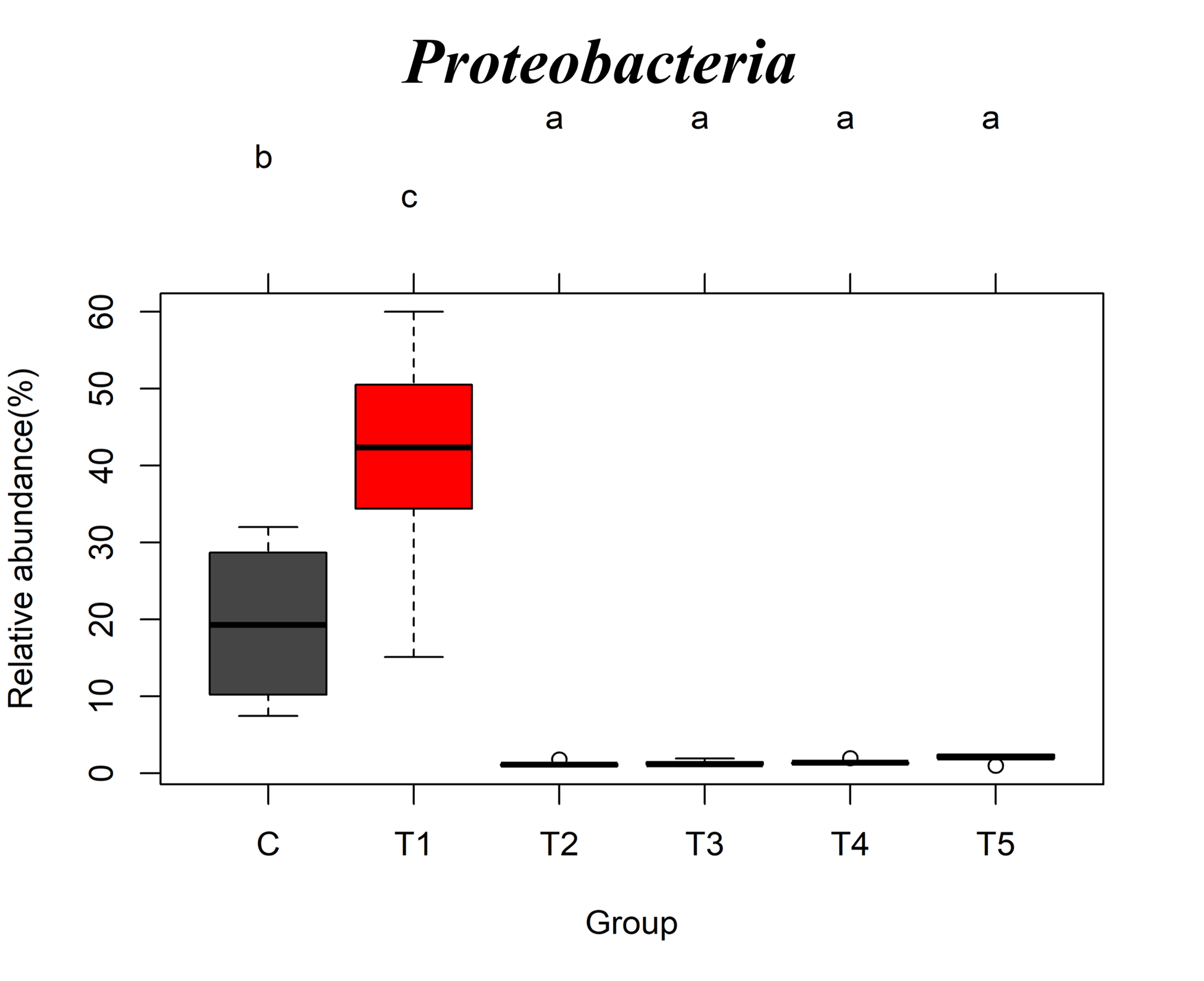

Supplement: Supplementary Figure 7 — Relative abundance of Proteobacteria by group. One-way ANOVA with Tukey’s post hoc test was used to determine significant differences among groups, and different superscript letters indicate a significant difference (p < 0.05). [file Image_7.TIF]

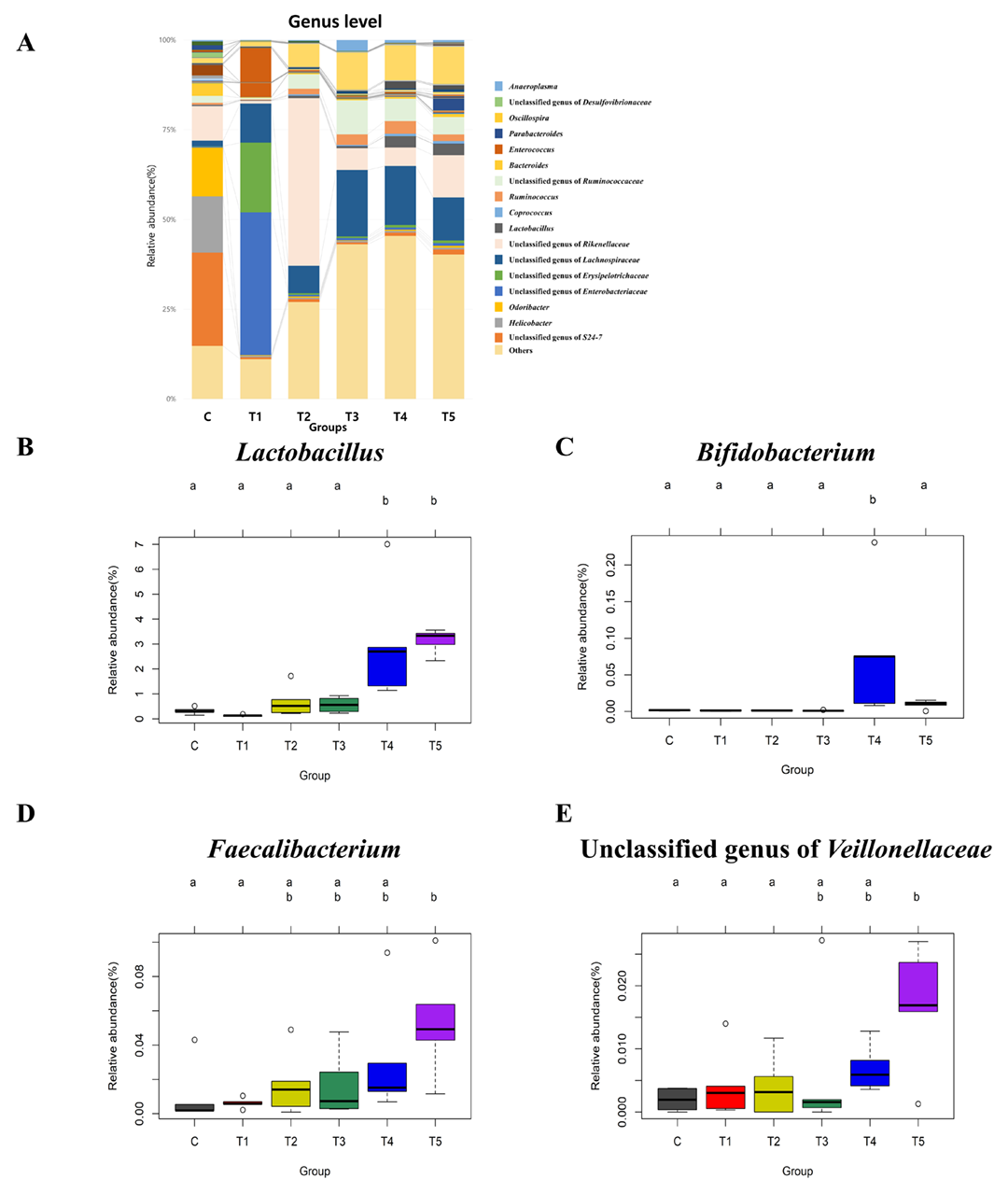

Supplement: Supplementary Figure 8 — Overall compositions of the gut microbiota at the genus level (A) and relative abundance of genera significantly differed by group. (B) One-way ANOVA with Tukey’s post hoc test was used for each genus to determine significant differences among groups, and different superscript letters indicate a significant difference (p < 0.05). [file Image_8.TIF]

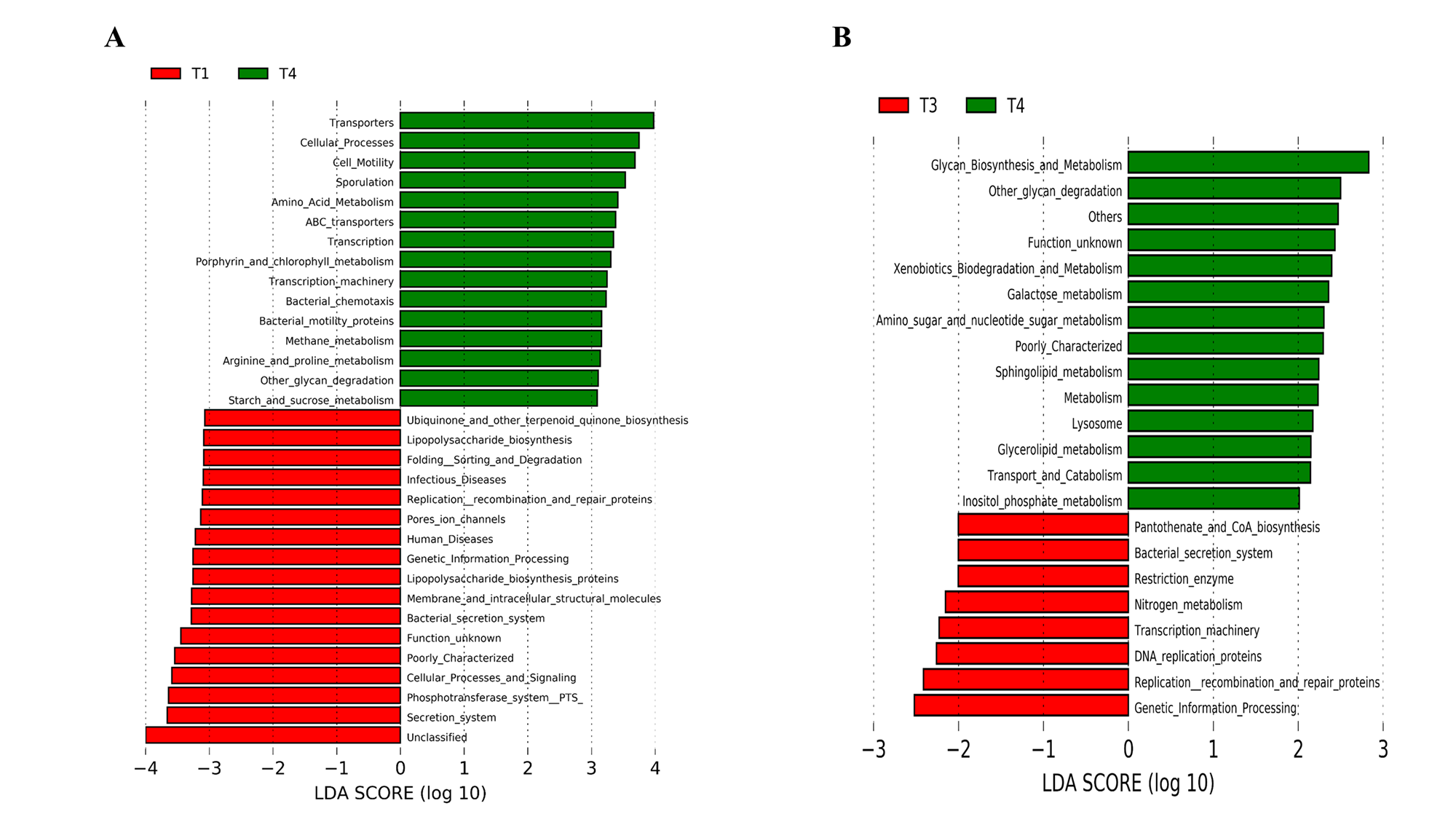

Supplement: Supplementary Figure 9 — Metagenomic prediction of gut microbiota of dysbiosis-induced mice after the trial to determine the effects of synbiotics LP/phthalyl pullulan nanoparticles (LP/PPN) (A; T1 vs. T4, linear discriminant analysis (LDA) score > 3.0) or prebiotics nanoparticulation (B; T3 vs. T4, LDA score > 2.0). Microbial functions were predicted using PICRUSt at the third level of the KEGG pathway, and LEfSe analysis was represented as histograms. [file Image_9.TIF]

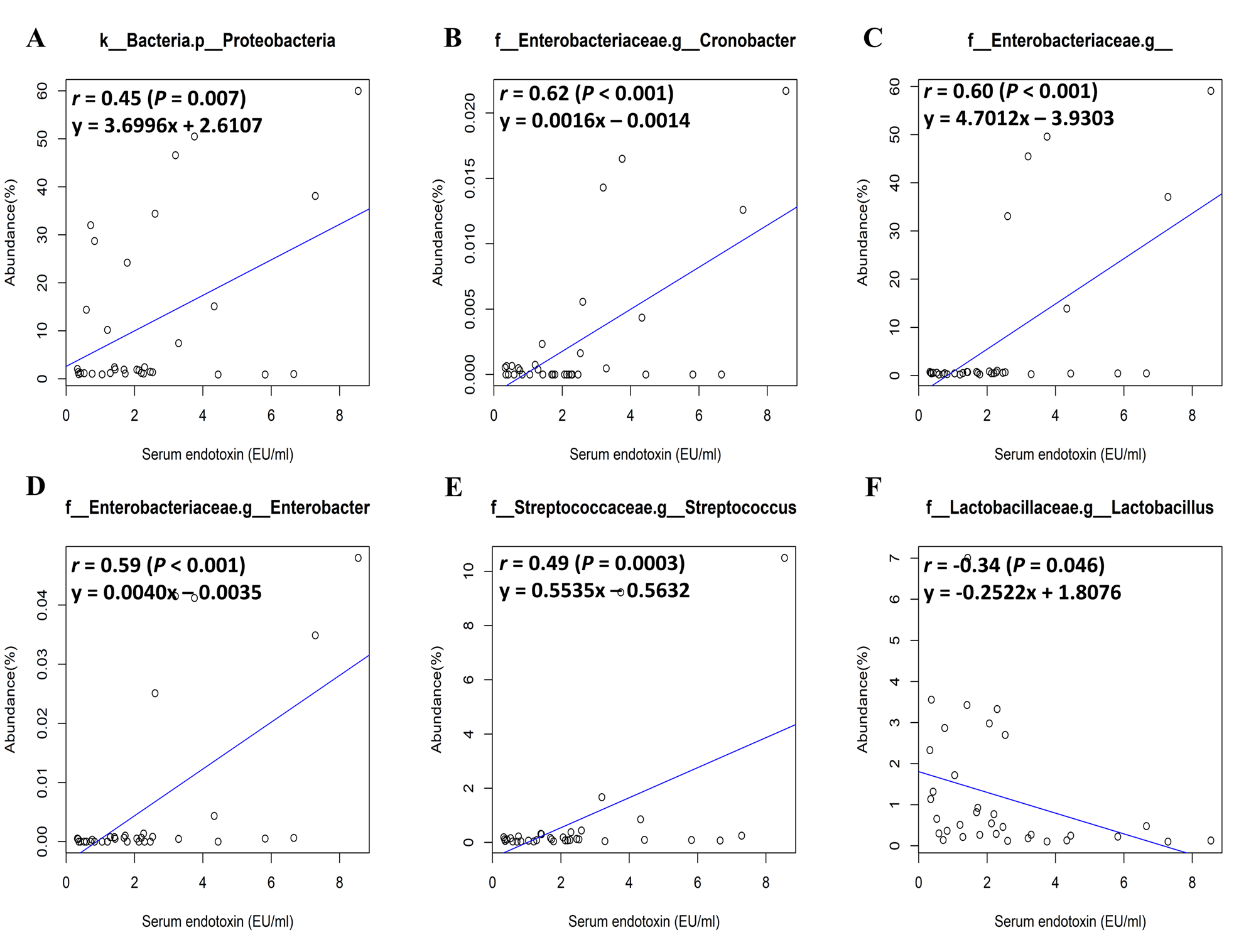

Supplement: Supplementary Figure 10 — Correlation analysis between the level of serum endotoxin and relative abundance of bacteria. Proteobacteria (A) was the only phylum that showed a correlation with serum endotoxin. Several genera, including Cronobacter (B), unclassified genus of the Enterobacteriaceae family (C), Enterobacter (D), and Streptococcus (E), were significantly positively correlated, whereas Lactobacillus (F) was significantly negatively correlated with the level of serum endotoxin. The relationship was assessed by Pearson’s correlation coefficient (r) and p-values from simple linear regression. [file Image_10.TIF]
